# Isolation and characterization of a halophilic *Modicisalibacter* sp. strain Wilcox from produced water

**DOI:** 10.1038/s41598-021-86196-0

**Published:** 2021-03-25

**Authors:** William S. Marsh, Brenden W. Heise, Mark J. Krzmarzick, Robert W. Murdoch, Babu Z. Fathepure

**Affiliations:** 1 Department of Microbiology and Molecular Genetics, Oklahoma State University, Stillwater, OK 74078 USA; 2 Civil and Environmental Engineering, Oklahoma State University, Stillwater, OK 74078 USA; 3 Center for Environmental Biotechnology, University of Tennessee, Knoxville, TN 37996 USA; 4 Battelle Memorial Institute, Columbus, OH 43201 USA

**Keywords:** Environmental microbiology, Microbiology, Environmental sciences

## Abstract

We report the isolation a halophilic bacterium that degrades both aromatic and aliphatic hydrocarbons as the sole sources of carbon at high salinity from produced water. Phylogenetic analysis of 16S rRNA-gene sequences shows the isolate is a close relative of *Modicisalibacter tunisiensis* isolated from an oil-field water in Tunisia. We designate our isolate as *Modicisalibacter* sp. strain Wilcox. Genome analysis of strain Wilcox revealed the presence of a repertoire of genes involved in the metabolism of aliphatic and aromatic hydrocarbons. Laboratory culture studies corroborated the predicted hydrocarbon degradation potential. The strain degraded benzene, toluene, ethylbenzene, and xylenes at salinities ranging from 0.016 to 4.0 M NaCl, with optimal degradation at 1 M NaCl. Also, the strain degraded phenol, benzoate, biphenyl and phenylacetate as the sole sources of carbon at 2.5 M NaCl. Among aliphatic compounds, the strain degraded n-decane and n-hexadecane as the sole sources of carbon at 2.5 M NaCl. Genome analysis also predicted the presence of many heavy metal resistance genes including genes for metal efflux pumps, transport proteins, and enzymatic detoxification. Overall, due to its ability to degrade many hydrocarbons and withstand high salt and heavy metals, strain Wilcox may prove useful for remediation of produced waters.

## Introduction

The inevitable contamination of soil and water by hydrocarbons and brine during oil and gas exploration, production and transportation operations results in potentially harmful impacts on human health and the environment^1^. For example, contamination of soil with saline wastewater (commonly called produced water (PW)) generated during oil and natural gas extraction can seriously damage soil properties, lead to soil erosion, cause the death of vegetation, and contaminate both surface and ground water^1,2^. Physicochemical treatment methods for the removal of hydrocarbons from PW are expensive and impractical. Hence, it is necessary to explore biological strategies for the remediation of PW. There have been numerous studies on the biodegradation of petroleum compounds in non-saline or low salinity conditions. Many microorganisms belonging to diverse genera have been isolated and their physiology, ecology, and genomics have been well characterized. In some instances, their practical applications have been realized^3–10^. However, relatively little is known about the microbial degradation of hydrocarbons in hypersaline environments such as PW, especially about their practical application for bioremediation. This is paramount, as conventional microorganisms cannot function well in high-salinity conditions due to disruption of the cell membrane, denaturation of proteins, and other detrimental effects.

Interest in the treatment of PW has significantly increased in recent years, as this is one of the oil-industry’s largest waste products generated; about 21 billion barrels of PW per year in the United States alone^11^. If properly treated, this water could be reused in agriculture or industry, especially considering recent droughts and water shortages in much of the south-central and western United States. Most PWs are highly saline, sometimes reaching > 30% (w/v) salinity (here and throughout the manuscript, we define salinity as the concentration of NaCl) and contain a variety of aromatic, alicyclic, and aliphatic hydrocarbons and other organics. In addition, a slew of heavy metals (Pb, Mg, Zn, Cu, Fe, B, and others) and naturally occurring radionuclides are often present in most PW^11^. These vast volumes of PW with unknown chemistries pose serious water management challenges and remediation is costly. To date, most oil and gas production operations dispose their PW via deep-well injection in Class II disposal wells^11,12^. However, such disposal practices have the potential to contaminate the subsurface and cause other environmental damage, including earthquakes^13,14^. Therefore, developing a cost-effective and safe bioremediation technology for the treatment of saline PW will not only prevent unintended seismic activity and contamination of subsurface aquifers but also help offset the shortage of freshwater resources.

In this article we report the isolation of a halophilic bacterium from PW from Payne County, OK, USA. This organism is capable of degrading both aliphatic and aromatic compounds at high salinity. Although there are recent reports on the isolation and characterization of microorganisms that degrade petroleum compounds from contaminated saline environments^15–20^, to our knowledge very little is known about similar microorganisms living in PW^21,22^. Therefore, understanding the diversity, ecology, physiology and catabolic potential of such organisms is crucial for developing technologies for the remediation of saline PW for beneficial uses.

## Results

### General features of the draft genome and the phylogeny of strain Wilcox

The strain Wilcox genome consists of 3,584,069 base pairs (bp) with an average G + C content of 67.25% and with 3283 predicted protein-coding genes. Single copies of 5S and 23S, and two copies of 16S rRNA genes, are present along with 53 tRNA genes in the genome.

**Figure 1:**
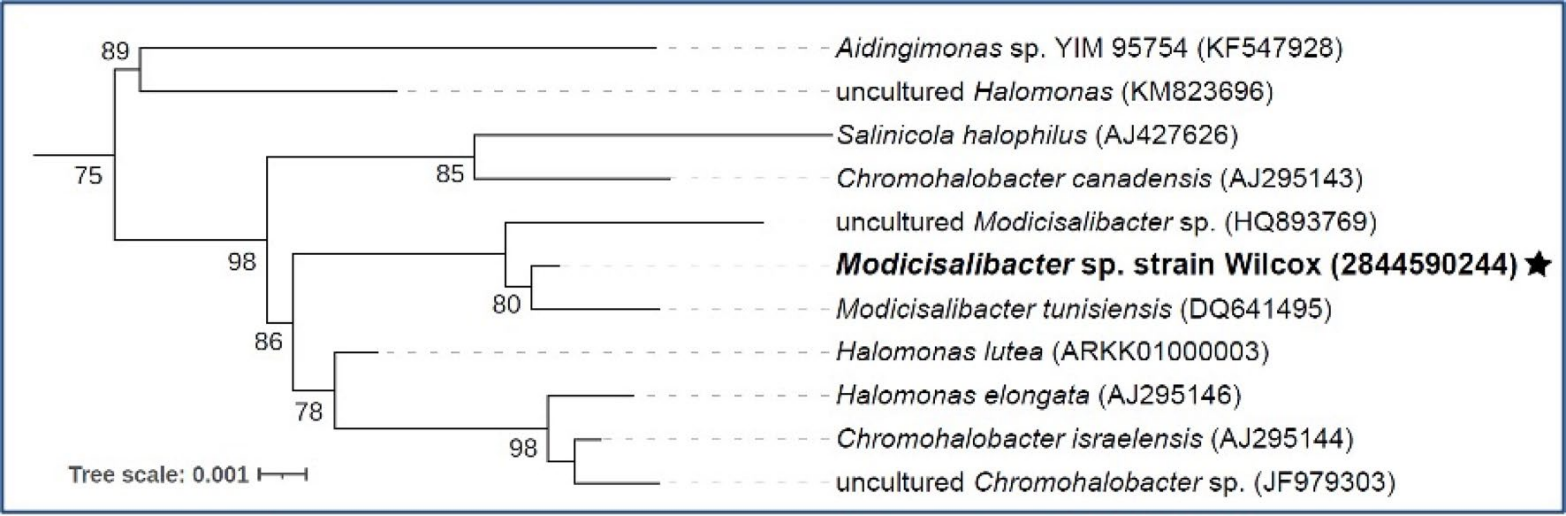
FastTree maximum likelihood approximation phylogenetic tree of 16S rRNA gene sequences of Wilcox and its most closely related phylogenetic neighbors. Only bootstrap values above 75% are shown. Integrated Microbial Genomes (IMG) Gene ID is provided for strain Wilcox, indicated by a star. Silva database accession numbers are included for all other sequences. The tree was constructed using the Alignment, Classification and Tree Service hosted at the SINA website^23^.

The isolate exhibited > 99% 16S rRNA -gene nucleotide identity to that of *Modicisalibacter tunisiensis* strain LIT2 (Class *Gammaproteobacteria*, Family *Halomonadaceae*) that was isolated from oil-field water in Tunisia^21^. The isolate also showed > 99% sequence identity to a 16S rRNA gene sequenced from an uncultured bacterium in San Juan Basin disposal site produced water (Silva accession number HQ893769). The members of the genus *Modicisalibacter* formed a distinct cluster from most closely related microbes in the genera *Halomonas, Chromohalobacter*, and *Salincola*, all members of family *Halomonadaceae* (Fig. 1)*.* The isolate is tentatively designated as *Modicisalibacter* sp. strain Wilcox.

### Prediction of hydrocarbon degrading genes in strain Wilcox

The genomic analysis of strain Wilcox provided the basis of hypotheses for hydrocarbon degradation mechanisms by the bacterium. Integrated Microbial Genomes (IMG) was the primary source of functional annotation information. We also used BLASTp to identify close homologs in the NCBI non-redundant protein database. Based on KEGG KO assignments and NCBI BLASTp search against NCBI nr, we predicted almost complete metabolic pathways for several aromatic compounds in silico in strain Wilcox genome (Table 1, Supplemental Table S2). Functional annotations suggested the presence of genes used for the metabolism of a variety of aromatic compounds and aliphatic compounds, including genes that code for oxygenases and dioxygenases such as phenol hydroxylase, salicylate hydroxylase, p-hydroxybezoate hydroxylase, 3-(3-hydroxy-phenyl) propionate/3-hydroxycinnamic acid hydroxylase, benzene 1,2-dioxygenasse, benzoate 1,2-dioxygenase, 2-halobenzoate 1,2-dioxygenase, and biphenyl dioxygenase alpha and beta subunits. These enzymes potentially catalyze the initial hydroxylation of a large variety of aromatic compounds into a few key central metabolic intermediates such as catechol, protocatechuate and gentisate^9,24–26^. In addition, genome analysis predicted most of the genes in the *paa* phenylacetate degradation pathway, by which phenylacetate is activated by a Co-A ligase and is then converted to ring-1, 2-epoxide by a multicomponent monooxygenase followed by a hydrolytic ring cleavage^27–29^. Interestingly, we did not find naphthalene and other polyaromatic hydrocarbon (PAH) related genes, such as genes that code for the upper pathway enzymes involved in the conversion of naphthalene to salicylate^30^.

**Table 1:** Putative functions and top homologs of *Modicisalibacter* sp. strain Wilcox genes predicted to encode proteins involved in metabolism of hydrocarbons.

IMG Gene ID^1^	Gene name	Putative function	Organism	% Identity	E-value	NCBI accession no
2844586937	*bphC*	Biphenyl-2,3-diol 1,2-dioxygenase	*Halomonas* sp. A11-A	92.6	0.00E + 00	WP_110068217.1
2844586938	*bphB*	Cis-2,3-dihydrobiphenyl-2,3-diol dehydrogenase	*Halomonas* sp. A11-A	94.93	0.00E + 00	WP_110068218.1
2844586939	*bnzD*	Benzene 1,2-dioxygenase system ferredoxin–NAD( +) reductase subunit	*Halomonas* sp. A11-A	89	0.00E + 00	WP_110068219.1
2844586941	*bphA2*	Biphenyl dioxygenase subunit beta	*Halomonas* sp. A11-A	84.95	2.00E−118	WP_110068221.1
2844586942	*bphA1*	Biphenyl dioxygenase subunit alpha	*Halomonas* sp. A11-A	94.3	0.00E + 00	WP_110068222.1
2844586958	*fadA*	Acetyltransferase	*Halomonas* sp. 362.1	81.94	2.00E−78	WP_129138321.1
2844587095	*ald*	Long-chain aldehyde dehydrogenase	*Halomonadaceae* bacterium T82-2	96	0.00E + 00	KXS36977.1
2844587430	*DODA*	4,5-DOPA dioxygenase extradiol	*Halomonas* sp. EAR18	86.04	8.00E−170	WP_136065726.1
2844587495	*nahG*	Salicylate hydroxylase	*Halomonadaceae* bacterium T82-2	88.25	0.00E + 00	KXS36864.1
2844587528	*alkT*	Rubredoxin-NAD( +) reductase	*Halomonadaceae* bacterium T82-3	99.74	0.00E + 00	KXS38198.1
2844588030	*menH*	2-succinyl-6-hydroxy-2,4-cyclohexadiene-1-carboxylate synthase	*Halomonadaceae* bacterium T82-2	95.83	0	KXS36894.1
2844588354	*fadA*	3-ketoacyl-CoA thiolase	*Halomonas xianhensis*	84.65	0.00E + 00	WP_134016041.1
2844588659	*pcaG*	Protocatechuate 3,4-dioxygenase alpha chain	*Halomonadaceae* bacterium T82-2	95.22	1.00E−145	KXS38033.1
2844588660	*pcaH*	Protocatechuate 3,4-dioxygenase beta chain	*Halomonadaceae* bacterium T82-2	99.18	2.00E−180	KXS38034.1
2844588668	*paaJ*	3-oxoadipyl-CoA/3-oxo-5,6-dehydrosuberyl-CoA thiolase	*Halomonadaceae* bacterium T82-2	97.51	0.00E + 00	KXS38042.1
2844588669	*pcaJ*	3-oxoadipate CoA-transferase subunit B	*Halomonadaceae* bacterium T82-2	99.22	0.00E + 00	KXS38043.1
2844588670	*pcaI*	3-oxoadipate CoA-transferase subunit A	*Halomonas* sp. 362.1	94.85	0.00E + 00	WP_129138993.1
2844588671	*pcaB*	3-carboxy-cis,cis-muconate cycloisomerase	*Halomonadaceae* bacterium T82-2	86.91	0.00E + 00	KXS38045.1
2844588678	*paaH*	3-hydroxyadipyl-CoA dehydrogenase	*Halomonas* sp. JS92-SW72	84.89	0.00E + 00	WP_119023241.1
2844588712	*nagL*	Maleylpyruvate isomerase	*Halomonadaceae* bacterium T82-2	87.27	1.00E−133	KXS38997.1
2844589229	*adh*	Alcohol dehydrogenase	*Halomonas* sp. EAR18	88.46	3.00E−178	WP_136069176
2844589416	*ahpC*	Alkyl hydroperoxide reductase C	*Halomonas coralii*	96.5	2.00E−140	WP_163649990.1
2844589672	*hmgA*	Homogentisate 1,2-dioxygenase	*Halomonadaceae* bacterium T82-2	95.86	0.00E + 00	KXS37722.1
2844589728	*paaE*	1,2-phenylacetyl-CoA epoxidase, subunit E	*Halomonas* sp. HAL1	70.47	0.00E + 00	WP_008957201.1
2844589730	*paaC*	1,2-phenylacetyl-CoA epoxidase, subunit C	*Halomonas* sp. JS92-SW72	79.92	1.00E−150	WP_119023124.1
2844589731	*paaB*	1,2-phenylacetyl-CoA epoxidase, subunit B	*Halomonas* bacterium	89.25	2.00E−57	WP_022521442.1
2844589732	*paaA*	1,2-phenylacetyl-CoA epoxidase, subunit A	unclassified *Halomonas*	90.99	0.00E + 00	WP_027958342.1
2844589733	*paaK*	Phenylacetate-coenzyme A ligase	*Halomonas pacifica*	83.52	0.00E + 00	WP_146804038.1
2844589734	*pcaF*	Beta-ketoadipyl-CoA thiolase	*Halomonas* sp. 3(2)	87.53	0.00E + 00	WP_151442119.1
2844589737	*paaG*	1,2-epoxyphenylacetyl-CoA isomerase	*Halomonas* sp. JS92-SW72	80.99	8.00E−156	WP_119023117.1
2844589738	*paaF*	2,3-dehydroadipyl-CoA hydratase	*Halomonas* sp. 3(2)	80.47	1.00E−144	WP_151442123.1
2844589751	*catB-1*	Muconate cycloisomerase 1	*Halomonadaceae* bacterium T82-2	94.1	1.00E−133	KXS37865.1
2844589752	*catB-2*	Muconate cycloisomerase 1	*Halomonas heilongjiangensis*	92	9.00E−63	PXX89496.1
2844589753	*catC*	Muconolactone Delta-isomerase	*Halomonadaceae* bacterium T82-2	97.92	3.00E−64	KXS37864.1
2844589754	*catA*	Catechol 1,2-dioxygenase	*Halomonas* nitroreducens	85.49	0	WP_126480973.1
2844589755	*benA*	2-halobenzoate 1,2-Fgenase large subunit	*Halomonadaceae* bacterium T82-2	100	1.00E−109	KXS37862.1
2844589756	*benB*	2-halobenzoate 1,2-dioxygenase small subunit	unclassified *Halomonas*	91.98	1.00E−109	WP_078089514.1
2844589757	*benC*	Benzoate 1,2-dioxygenase electron transfer component	unclassified *Halomonas*	91.98	0	WP_078089514.1
2844589758	*benD*	1,6-dihydroxycyclohexa-2,4-diene-1-carboxylate dehydrogenase	*Halomonas aestuarii*	85.6	1.50E−159	WP_163650312.1
2844589848	*pobA*	p-hydroxybenzoate hydroxylase	*Halomonas coralii*	92.64	0	WP_129140614.1
2844589969	*fadI*	Acetyl-CoA acetyltransferase	*Halomonadaceae* bacterium T82-2	98.47	0.00E + 00	KXS37177.1
2844590194	*mphP*	Phenol hydroxylase P5 protein	*Marinobacter pelagius*	80.73	7.00E−47	WP_113861973.1

^1^Gene sequences were BLAST p searched against NCBI nr database in NCBI for top homologs.

The genome analysis predicted genes for aromatic ring cleavage enzymes such catechol 1,2-dioxygenase and protocatechuate 3,4-dioxygenase, two of the most common aromatic ring cleaving enzymes that produce oxygenolytic ring-cleavage products that enter central metabolism via beta-ketoadipate pathway^9,31^. Most of the genes in beta-ketoadipate pathway were present on the genome (Table 1 and Supplemental Table S2).

In addition, the genome of strain Wilcox harbors some of the genes needed for the degradation of n-alkanes. For example, we detected the gene that codes for rubredoxin-NAD (+) reductase, one of the three components of alkane hydroxylase system (AlkB). In addition, analysis predicted the presence of alcohol dehydrogenase and aldehyde dehydrogenase, suggesting the organism’s potential to metabolize n-alkanes through oxidation of the terminal methyl group to the corresponding primary alcohol and then to fatty acids, which enter beta-oxidation steps for complete metabolism. While we did not find a homolog of alkane monooxygenase of the AlkB complex, we surmise the presence of other genes, possibly a novel gene or mechanism responsible for the initial oxidation of n-alkanes to the corresponding alcohols.

### Genome prediction of heavy metal resistance in strain Wilcox

Microorganisms possess various resistance mechanisms against toxic heavy metals such as active efflux systems, bioaccumulation and enzymatic reduction^32^. Produced water are naturally enriched with a variety of heavy metals depending on the formation geology, age of the oil well, and oil extraction activities^33^. Genome analysis indicated the presence of a collection of putative metal-exporting and other resistance systems that might facilitate the survival of strain Wilcox in heavy metals containing PW, including several P-type ATPases, efflux pumps, antiporters, and transport proteins and other mechanisms responsible for removing metal ions out of the cells (Table 2 and Supplemental Table S3). We found genes that code for P-type ATPases for copper, zinc, cadmium, lead, manganese and efflux pumps for manganese and cobalt in the genome. P-type ATPases comprise a large protein family that pump out a variety of heavy metal ions and lipids across cellular membranes^34,35^. We predicted arsenic detoxification genes *arsC_1*, *arsC_2*, and *arsC_3* that code for arsenate reductases in strain Wilcox’s genome. In addition, we found genes for arsenical pump membrane protein ArsB and arsenical-resistance protein Acr3. These systems catalyze reduction of As (V) to As (III) by arsenate reductase which then can be expelled from the cell by an efflux pump^36,37^. Similarly, mercuric reductase gene, *merA* was found on the genome. Mercuric reductase reduces ionic mercury (Hg^2+^) into the less toxic metallic mercury (Hg^o^) form, which then evaporates^38,39^. Overall, strain Wilcox is endowed with robust heavy metal resistance system.

**Table 2:** Putative functions and top homologs of *Modicisalibacter* sp. strain Wilcox genes predicted to encode proteins involved in heavy metal resistance.

IMG Gene ID^1^	Gene name	Putative Function	Organism	% Identity	E-value	NCBI accession no
2844587032	*acr3*	Arsenical-resistance protein	*Halomonas xianhensis*	100	0	WP_092850561.1
2844587034	*arsC_1*	Arsenate reductase	*Halomonas xianhensis*	100	9.00E−95	WP_092850563.1
2844587035	*arsC_2*	Protein ArsC	*Halomonas xianhensis*	100	8.00E−98	WP_092850567.1
2844587328	*czcD*	Cadmium, cobalt and zinc/H( +)-K( +) antiporter	*Halomonadaceae bacterium* T82-2	98.73	0.00E + 00	KXS37941.1
2844587374	*mntB*	Manganese transport system membrane protein	*Halomonadaceae bacterium* T82-2	94.61	0.00E + 00	KXS36360.1
2844587477	*merA*	Mercuric reductase	*Marinobacter daqiaonensis*	91	0	WP_092012523.1
2844587480	*copA_1*	Copper-exporting P-type ATPase	*Marinobacter salarius*	94.67	0.00E + 00	WP_085682201.1
2844587740	*arsA*	Arsenical pump-driving ATPase	*Halomonadaceae bacterium* T82-2	100	0	KXS39846.1
2844587781	*copA_2*	Copper-exporting P-type ATPase	*Halomonadaceae bacterium* T82-2	93.61	0.00E + 00	KXS39808.1
2844587847	*copA_3*	Copper-exporting P-type ATPase	*Halomonadaceae bacterium* T82-2	91.27	0.00E + 00	KXS39748.1
2844587870	*mntP*	putative manganese efflux pump	*Halomonas* sp. 362.1	83.07	2.00E−99	WP_129141166.1
2844588193	*zur*	Zinc uptake regulation protein	*Halomonadaceae bacterium* T82-2	96	7.00E−99	KXS39292.1
2844588553	*zntB*	Zinc transport protein	*Halomonadaceae bacterium* T82-2	98.5	0.00E + 00	KXS38423.1
2844588586	*zntA*	Zinc/cadmium/lead-transporting P-type ATPase	*Halomonadaceae bacterium* T82-2	97.4	0.00E + 00	KXS38456.1
2844589668	*arsB*	Arsenical pump membrane protein	*Halomonas* sp. SL1	87.82	0	WP_107181297.1
2844589669	*arsC_3*	Arsenate reductase	*Halomonadaceae bacterium* T82-2	91.18	8.00E−84	KXS37725.1
2844589689	*ctpC*	putative manganese/zinc-exporting P-type ATPase	*Halomonadaceae bacterium* T82-2	94.96	0.00E + 00	KXS37705.1
2844589846	*mco*	Multicopper oxidase	*Halomonadaceae bacterium* T82-2	99.57	0.00E + 00	KXS37229.1
2844589924	*corC*	Magnesium and cobalt efflux protein CorC	*Halomonadaceae bacterium* T82-2	98.97	1.00E−146	KXS39516.1
2844590090	*cbiX*	Sirohydrochlorin cobaltochelatase	*Halomonadaceae bacterium* T82-2	91.27	1.00E−74	KXS38081.1

^1^Gene sequences were BLAST p searched against NCBI nr database in NCBI for top homologs.

### Genome-guided laboratory validation of the hydrocarbon degradation capacity of strain Wilcox

The genome analysis of strain Wilcox predicted a repertoire of hydrocarbon degrading genes. Genome-guided laboratory culture studies were initiated to corroborate the genome predicted catabolic potential of strain Wilcox to degrade important hydrocarbons. Table 3 shows the strain’s ability to degrade both monoaromatic hydrocarbons (MAH), biphenyls, PAHs as well as n-alkanes. Our culture studies revealed that strain Wilcox is capable of degrading all tested MAH such as benzene, toluene, ethylbenzene, and xylenes (BTEX) as well as, phenol, benzoate, biphenyl, and phenylacetate as the sole sources of carbon at high salinity. However, no degradation of PAHs such as naphthalene and phenanthrene occurred even after four weeks of incubation under similar growth conditions. Strain Wilcox was also capable of degrading n-decane and n-hexadecane as the sole sources of carbon and energy at high salinity (Supplemental Fig. S1 and S2). However, no apparent degradation of smaller-chain alkanes such as methane and hexane or of longer-chain alkanes such as n-eicosane and n-dotriacontane occurred after four weeks of incubation under similar growth conditions.

**Table 3:** Laboratory assessment of the biodegradation ability of strain Wilcox.

Hydrocarbon class	Hydrocarbons tested as substrate ^a^	Genome predicted degradation	Experimental validation of biodegradation
MAH	Benzene (20–40 µmol/bottle)	** + **	** + **
	Toluene (20–40 µmol/bottle)	** + **	** + **
	Ethylbenzene (20 -30 µmol/bottle)	** + **	** + **
	Xylenes (20–30 µmol/bottle)	** + **	** + **
	BTEX (combined)	** + **	** + **
	Phenol (2 mM)	** + **	** + **
	Benzoate (1–2 mM)	** + **	** + **
	Phenylacetate (2 mM)	** + **	** + **
Biphenyl	Biphenyl (2 mM)	** + **	** + **
PAH	Naphthalene (1–2 mM)	** − **	** − **
	Phenanthrene (1–2 mM)	** − **	** − **
n-Alkanes	Methane (100 uM)	** − **	** − **
	Hexane (5 mM)	** − **	** − **
	Decane (2 mM)	** + **	** + **
	Hexadecane (5 mM)	** + **	** + **
	Eicosane (2 mM)	** − **^**b**^	** − **
	Dotriacontane (2 mM)	** − **^**b**^	** − **

Strain Wilcox grown in mineral salts medium supplemented with 2.5 M NaCl and amended with a hydrocarbon as the sole source of carbon and energy were used to study the degradation potential of strain Wilcox. Cultures were inoculated with approximately 5 × 10^5^ or 1.2 × 10^6^ CFU of strain Wilcox.

^ a^BTEX compounds and methane were determined using a gas chromatograph^40,41^. Degradation activities on biphenyl, phenol, phenylacetate, n-hexane, n-decane, n-hexadecane, n-eicosane, and n-dotriacontane were determined as CFU. Benzoate degradation was monitored using absorption at 223 λ using a spectrometer.

^**b**^We did not identify homologs of *almA* or *ladA* genes known to act on longer-chain alkanes. ( +) Degraded as the sole carbon and energy source. ( −) No growth or degradation. The data points are averages of triplicate bottles.

**Figure 2:**
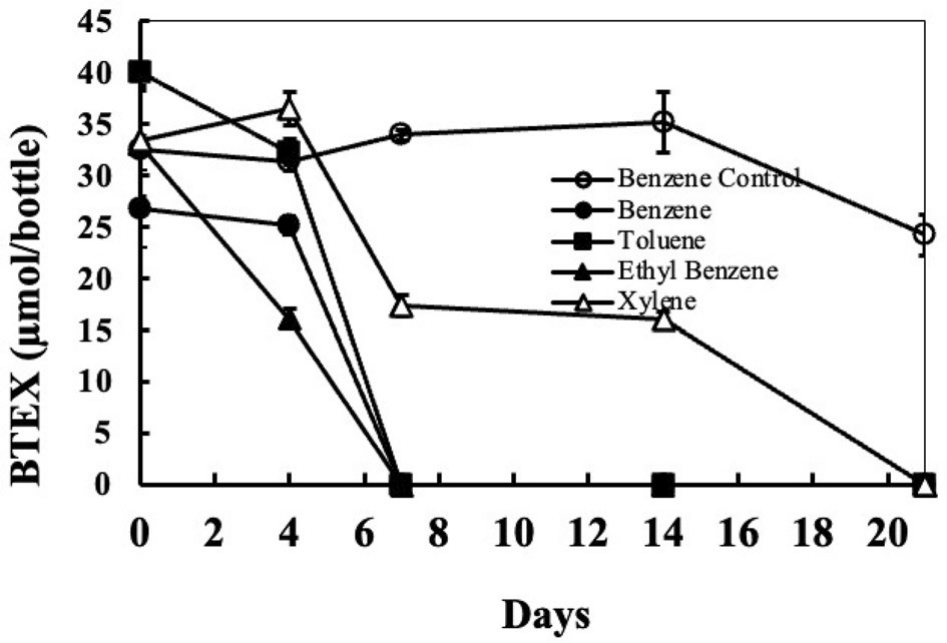
The profile of BTEX degradation in MSM containing 2 M NaCl. Cultures containing 49 ml of MSM supplemented with 2 M NaCl were set up. Cultures were spiked with BTEX and inoculated with 1 ml (5 × 10^5^ CFU) of strain Wilcox. Culture bottles were closed with Teflon-coated septa and aluminum crimps. Un-inoculated controls were prepared similarly (only benzene is shown). BTEX concentrations were determined by injecting 100 µL of headspace sample into a gas chromatograph (GC) at given time points. Error bars indicate ± 1 standard deviation of triplicate bottles (n = 3).

Figure 2 shows degradation kinetics of BTEX compounds in mineral salts medium (MSM) supplemented with 2 M NaCl^42^. Complete degradation of benzene, toluene, and ethylbenzene occurred in 7 days at a rate of approximately 4–6 µmole/day. Degradation of xylene proceeded after a lag of 4 days and took almost 17 days thereafter for complete mineralization at a rate of roughly 2.15 µmole/day. These results clearly show the ability of strain Wilcox to degrade some of the most problematic hydrocarbons.

**Figure 3:**
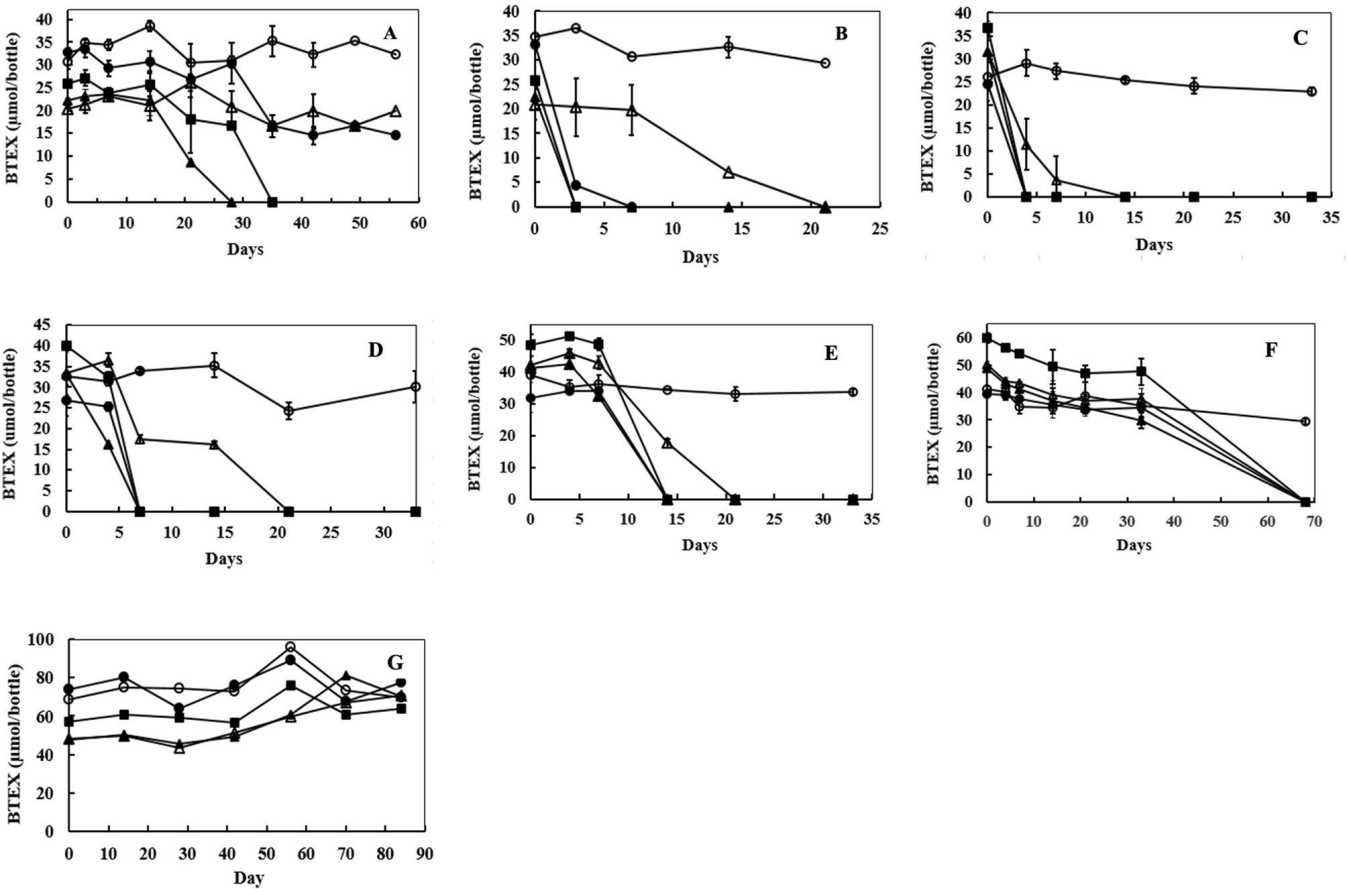
Degradation of BTEX by strain Wilcox at varied salt concentrations. Culture bottles contained 48 ml MSM supplemented with (**A**), 0.016 M NaCl; (**B**), 0.5 M NaCl; (**C**), 1.0 M NaCl; (**D**), 2.0 M NaCl; (**E**), 3.0 M NaCl; (**F**), 4.0 M NaCl or (**G**), 5.0 M NaCl. Cultures were inoculated with 2 ml (approximately 1.2 × 10^6^ CFU) of strain Wilcox culture and closed with Teflon-coated septa and aluminum crimps. Un-inoculated controls were prepared similarly (only data for benzene is shown). Relatively higher amounts of BTEX were measured in the headspace in bottles with high salt concentrations (4 and 5 M NaCl) suggesting lower solubility of these compounds at higher salinity. Symbols: Benzene Control (− ○ −); Benzene (− ● −); Toluene (− ■ −); Ethylbenzene (− ▲ −); and Xylenes (− △ −). Error bars indicate ± 1 standard deviation of triplicate bottles (n = 3).

We evaluated the strain’s ability to degrade BTEX in MSM at salt concentrations ranging from 0.016 M to 5 M NaCl (Fig. 3). Complete degradation of BTEX occurred in the presence of 0.5 to 4 M NaCl (Fig. 3B–F) with most rapid degradation at 1 M NaCl. Interestingly, as seen in Fig. 3A, degradation proceeded at much slower rates in the presence of the lowest concentration of NaCl (0.016 M NaCl). Ethylbenzene and toluene were completely degraded in 4 and 5 weeks, but little degradation of benzene or xylene was seen during the same time periods suggesting perhaps different enzymes may be responsible for the degradation of toluene and ethylbenzene compared to benzene and xylenes. BTEX degradation in the presence of 4 M NaCl (Fig. 3F) was slow, requiring 70 days for complete degradation, while no degradation occurred in the presence of 5 M NaCl even after incubating for > 12 weeks (Fig. 3G). To determine the viability of cells after 12 weeks of incubation at 5 M NaCl, we plated these cells on agar plates prepared with 5 mM acetate and 1 M NaCl. No colonies appeared on the plates, suggesting cells were completely killed at 5 M NaCl.

**Figure 4:**
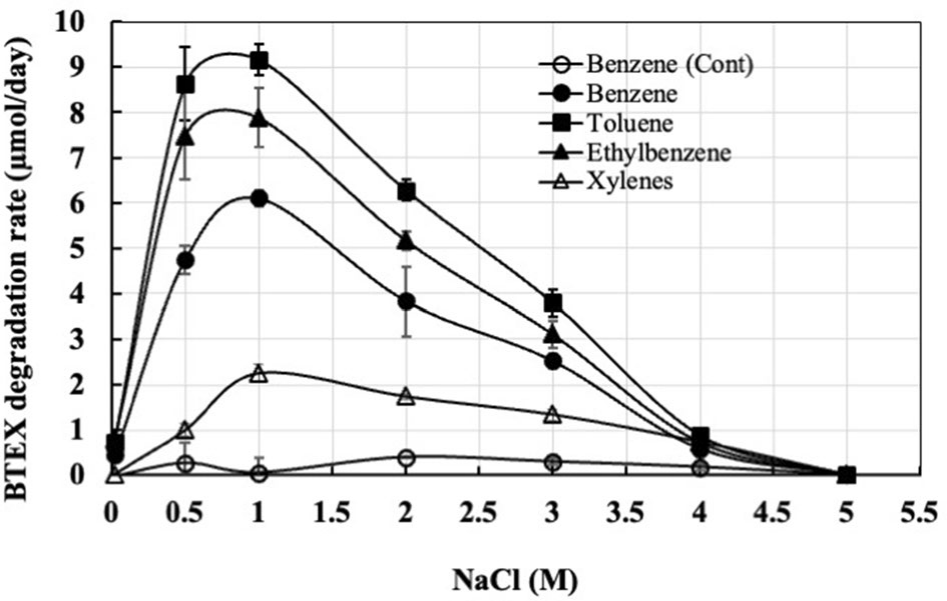
BTEX degradation rates in the presence of different salt (NaCl) concentration. Optimal degradation of BTEX degradation occurred at 1 M NaCl. The rates of BTEX compounds increased initially reaching maximum at 1 M NaCl and the rate decreased progressively with increasing NaCl concentration. No degradation of BTEX occurred in bottles containing 5 M NaCl over 12 weeks of incubation. Error bars indicate ± 1 standard deviation of triplicate bottles (n = 3).

Figure 4 shows the rate of BTEX degradation at varied salt concentrations. Results show that among BTEX, toluene was degraded most rapidly (9.2 µmol/day) at all NaCl concentrations, followed by ethylbenzene (8 µmol/day), benzene (6.1 µmol/day), and xylenes (2.25 µmol/day). In addition to BTEX, strain Wilcox also utilizes benzoate, phenol, biphenyl, and phenylacetate as the sole sources of carbon and energy at 2.5 M NaCl. Complete degradation of benzoate occurred in 4 weeks and no degradation benzoate occurred in un-inoculated flasks (Supplemental Fig. S3). Complete degradation of benzoate took longer than any other aromatic compound tested, which might be due to toxic nature of benzoate. Growth on phenol, biphenyl, and phenylacetate was determined as CFU and results show an increase in cell numbers by at least 3–4 log units from an initial 10^3^ or 10^4^ CFU/ml within one to two weeks (Supplemental Fig. S4, S5, and S6).

### Bioaugmentation of PW by strain Wilcox

**Figure 5:**
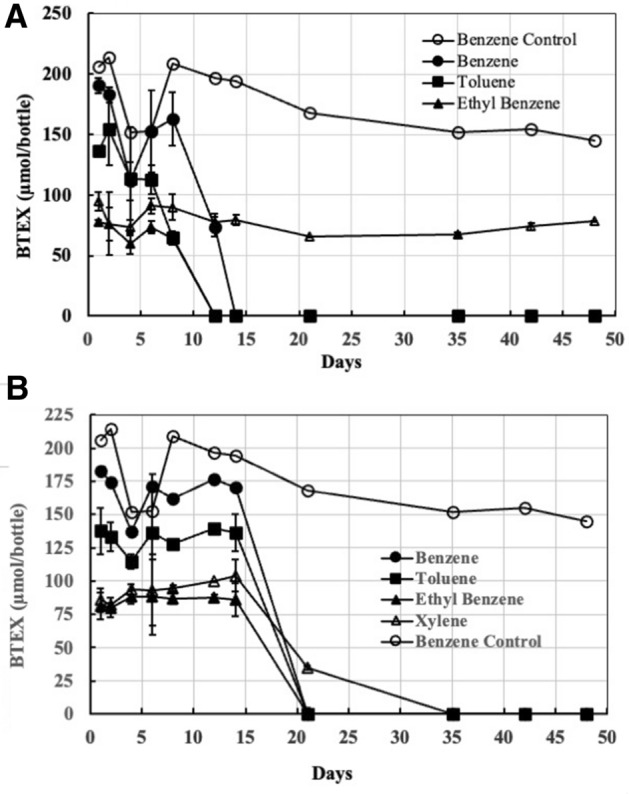
Degradation of BTEX in PW (no nutrients except the added hydrocarbons) as the sole source (s) of carbon amended individually (**A**) and together (**B**). Microcosms containing 48 ml of formation PW were inoculated with 2 ml (roughly 1.2 × 10^6^ CFU) of strain Wilcox and closed with Teflon-coated septa and aluminum crimps. Autoclaved control microcosms amended with benzene, toluene, ethylbenzene and/or xylenes were prepared similarly (only benzene data is shown). Degradation of BTEX was monitored at time points by injecting 100 µL headspace samples into onto a GC. Error bars indicate ± 1 standard deviation of triplicate bottles (n = 3).

We assessed strain Wilcox’s ability to survive and degrade amended BTEX compounds directly in PW collected from the First Wilcox formation in Payne County, OK. Results (Fig. 5) show the strain’s ability to degrade BTEX compounds when provided individually or as a mixture. The organism degraded benzene, toluene or ethylbenzene as the sole source carbon within 12–14 days and no degradation of added xylene occurred even after incubating for 48 days (Fig. 5A). No or little degradation of added hydrocarbons occurred in control bottles containing PW not bioaugmented with strain Wilcox. These results clearly show the organism’s potential to degrade hydrocarbons in PW at high concentrations. Degradation proceeded after a longer lag time when BTEX compounds were added together (Fig. 5B). Complete degradation of benzene, toluene, and ethylbenzene occurred in 21 days compared to 12–14 day when these compounds were fed individually. The longer lag time could be due to overall higher concentrations of BTEX when fed together. Interestingly, added xylene was completely degraded in 35 days when BTEX were fed together compared to when these compounds were fed individually (Fig. 5A), suggesting that xylene is degraded by enzymes induced by the presence of benzene, toluene, and/or ethylbenzene and is not able to induce for its own metabolism.

## Discussion

Large amounts of PWs are generated each year during oil and gas production operations and these wastewaters with complex physio-chemical properties pose a serious water management challenges and remediation is costly^43^. There is great interest in developing promising biological strategies for the remediation and reuse of PW after desalination, especially in the South-central and Western United States, where water scarcity has become a serious concern.

Biodegradation of hydrocarbons in PW is difficult or even impossible by traditional non-halophilic microorganisms due to high salinity and other toxicities. Halophiles or halotolerant microorganisms with hydrocarbon degradation capacity, on the other hand, are promising candidates for the bioremediation of hydrocarbons in PW. Recent studies have shown the biodegradation potential of halophiles and halotolerant bacteria and archaea in contaminated saline environments such as oilfields, (semi)-arid coastal regions, salt lakes, sabkhas, and others^15–20,44^. However, such information is seriously lacking for PW, despite the staggeringly large volumes of highly saline PW generated all over the world. Therefore, studies are urgently needed that explore microbes that can survive high salinity and able to mineralize petroleum compounds in PW.

### Genome prediction of major hydrocarbon degradation pathways in Strain Wilcox

In this article, we report the isolation and characterization of a halophilic bacterium, *Modicisalibacter* sp. strain Wilcox from PW that is capable of degrading a variety of petroleum compounds at high salinity. We predicted in silico nearly complete metabolic pathways for a number of hydrocarbons in strain Wilcox genome. For example, we detected genes for several monooxygenases and dioxygenases that hydroxylate aromatic compounds to a few common intermediates such as catechol, protocatechuate, and gentisate^9,24–26^. Also, we predicted genes for ring cleavage enzymes such as catechol 1,2 dioxygenase and protocatechuate 3,4-dioxygenase that produce dearomatized products that are further degraded by the genes in the beta-ketoadipate pathway to central metabolites that enter the TCA cycle^9,31^ (Table 1). In addition, genome analysis predicted most of the genes in the *paa* phenylacetate degradation pathway that combines steps from aerobic and anaerobic catabolism of aromatic compounds. Genome analysis also predicted genes needed for the oxidation of medium-chain n-alkanes to the corresponding fatty acid that are further oxidized by beta-oxidation. These diverse and complete pathways suggest strain Wilcox has broad degradation abilities and is well suited for biodegradation of complex hydrocarbon mixtures at high salinity. These observations are consistent with other halophiles isolated from a variety of high salinity environments. (15–20).

At low concentrations, heavy metals are essential for cell survival and function, but become toxic when present at high concentration^39^. Microorganisms possess various resistance mechanisms against toxic heavy metals such as active efflux, bioaccumulation, and enzymatic reduction^32^. Many metal ions such as lead, cadmium, mercury, and aluminum have no biological role and exert inhibitory effects on cells by replacing essential metals from their natural binding sites, disrupting cell membranes, altering enzyme specificity, and denaturing DNA^32,39^. PWs are naturally enriched with a variety of heavy metals depending on the formation geology, age of the oil well, and oil extraction activities^33^. Genome analysis indicated the presence of a collection of putative metal-exporting and other resistance systems that might facilitate the survival of strain Wilcox in PW containing heavy metals. For example, we found genes that code for several P-type ATPases, efflux pumps, antiporters, transport proteins, and other mechanisms responsible for removing metal ions (Table 2). The genome analysis also predicted genes that code for arsenic and mercuric resistance systems. Altogether, these results indicate that strain Wilcox is endowed with robust heavy metal resistance capabilities. It is not surprising to find the presence of different mechanisms to cope with several heavy metals in strain Wilcox because PW are rich in a variety of heavy metals, some at high concentrations presumably from crude oil^45,46^. These findings are consistent with other halophiles that possess robust metal resistance mechanisms, as these organisms live in hypersaline environment where a variety of heavy metals accumulate due to agriculture, mining, industrial activities^47^.

### Laboratory validation of genome-predicted hydrocarbon degradation potential in strain Wilcox

Laboratory cultures studies confirmed that the strain was able to completely mineralize BTEX within 7 days in MSM at 2 M NaCl with no lag time, with the exception of xylene, which took almost 21 days (Fig. 2). We further assessed the strain’s ability to degrade BTEX in the presence of various salt concentrations (Fig. 3). These laboratory studies showed that the strain was able to degrade BTEX over a wide range of salinity ranging from 0.016 to 4 M NaCl, with optimum degradation at 1 M NaCl. No degradation of BTEX occurred at 5 M NaCl even after 3 months of incubation. When we plated cells from these cultures, no colonies appeared, suggesting complete death of strain Wilcox at 5 M NaCl. Degradation proceeded at much slower rates under low salinity (0.016 M NaCl), suggesting strain Wilcox is a true halophile that requires high salinity for growth. Previous studies have shown both negative and positive impacts of salt on hydrocarbon degradation ability by microorganisms^18^. Estimation of the rate of BTEX degradation by strain Wilcox at different salinities suggest that toluene was degraded fastest, followed by ethylbenzene, benzene, and xylenes when fed together (Fig. 4). We found that strain Wilcox degraded BTEX at maximal rates at 1 M NaCl, and the rate progressively decreased with increasing salt concentration. Previous studies using strain Seminole grown under similar conditions showed that strain Seminole degraded benzene optimally at a rate of 2.5 µmol/bottle compared to 6 µmol/bottle by strain Wilcox suggesting superior rate by strain Wilcox^48^. The strain’s ability to degrade BTEX over a wide salinity range could be beneficial, as PW contain varying levels of salinities depending on the source and geology. Strain Wilcox also degraded other aromatic compounds such as phenol, biphenyl, benzoate, and phenylacetate, which is important, as most PW contain a variety of aromatic compounds along with BTEX, which are more problematic than many other hydrocarbons. Similar observations have been made in many halophilic and halotolerant hydrocarbon-degrading microorganisms from contaminated high salinity environments^16,17,19,44,48,49^.

Genome analysis predicted the presence of *mphP* and *bnzD* genes that code for ring hydroxylating monooxygenases and dioxygenases such as phenol hydroxylase and benzene 1,2-dioxygenase, respectively. We surmise that these mono and dioxygenases may be responsible for the initial hydroxylation of benzene, toluene, ethylbenzene, xylenes, and phenol to central metabolites such as catechol^50,51^. The strain also degrades benzoate as the sole source of carbon, which is consistent with the presence of corresponding benzoate metabolic genes such as *benABC*, which code for a benzoate 1,2-dioxygenase complex that converts benzoate to *cis*-1,6-dihydroxy-2,4-cyclohexadiene-1-carboxylic acid which is then converted to catechol by 1,6-dihydroxycyclohexa-2,4-diene-1-carboxylate dehydrogenase coded by *benD* gene^52^.

Strain Wilcox rapidly degraded biphenyl when presented as sole carbon and energy source. Biphenyl degradation is consistent with the presence of genes needed for the upper biphenyl degradation steps. We found homologs of *bphA1* and *bphA2*, which code for biphenyl dioxygenase alpha and beta subunits that catalyze the oxidation of biphenyl to a dihydrodiol compound (2,3-dihydroxy-4-phenylhexa-4,6-diene) which is then converted to a dihydroxy compound (2,3-dihydroxybiphenyl) by *cis*-2,3-dihydrobiphenyl-2,3-diol dehydrogenase encoded by *bphB*, also detected on the genome. The dihydroxy-compound is then degraded to 2-hydroxy-6-oxo-6-phenylhexa-2, 4-dienoic acid by the ring-cleavage dioxygenase biphenyl-2,3-diol 1,2-dioxygenase encoded by *bphC* gene^53^. We did not detect a *bphD* gene homolog in strain Wilcox genome, which is responsible for the final conversion of the ring-cleavage product to benzoic acid and 2-hydroxypenta-2, 4-dienoic acid. We hypothesize this step may be catalyzed by other genes in the genome and benzoate formed in this step can be further oxidized to catechol by benzoate 1,2-dioxygenase encoded by *benABC* and 1,6-dihydroxycyclohexa-2,4-diene-1-carboxylate dehydrogenase encoded by *benD*, each of which were detected in the genome^52^.

Catechol produced from BTEX, phenol, benzoate and biphenyl can likely undergo ring cleavage by catechol 1,2-dioxygenase, encoded by the *catA* gene found on the genome. The ring cleavage compounds are further degraded by genes in the catechol branch of the beta-ketoadipate pathway^9,31^. Most of these genes needed for the downstream steps are predicted to be present in strain Wilcox genome (Table 1 and Supplemental Table S2).

Strain Wilcox also degrades phenylacetate in accordance with the presence of requisite genes on the genome. The gene *paaK* codes for phenylacetate-CoA ligase that converts phenylacetate to phenylacetate-CoA which undergoes ring epoxidation by a multicomponent monooxygenase coded by a *paaABCE* gene cluster. The reactive and unstable epoxide is further isomerized to oxepin-CoA by the ring-1,2-epoxyphenylacetyl-CoA isomerase coded by *paaG* gene. This is followed by hydrolytic cleavage and beta-oxidation-like steps yielding acetyl-CoA and succinyl-CoA that enter TCA cycle^29^. We predicted most of the genes needed for these downstream steps (*paaJGFH*) in the genome, except the gene that code for hydrolytic cleavage of oxepin-CoA (*paaZ*). We assume this step might be catalyzed by other enzymes in strain Wilcox^29^. The presence of the *paa* gene cluster responsible for phenylacetate degradation also suggests the organism’s potential to metabolize similar compounds such as lignin-derived aromatic compounds as well environmental pollutants like styrene and ethylbenzene. The *paa* pathway is also utilized by *Escherichia coli*, *Pseudomonas putida,* other bacteria^28,29,54^ including halophiles such as *Arhodomonas* sp. strain Seminole^48^ and species of the genus *Halomonas*^49^.

Among the tested aromatic compounds, strain Wilcox did not degrade naphthalene or phenanthrene even after incubating for > 4 weeks. The lack of degradation of naphthalene and phenanthrene is consistent with the lack of genes for the degradation of PAHs such as naphthalene 1,2-dioxygenase and PAH ring-hydroxylating dioxygenases (PAH-RHD), respectively, that catalyze the initial oxidation of naphthalene and PAHs^55^. Interestingly, a phylogenetically closely related organism isolated from a produced water treatment plant in Brazil, *Modicisalibacter tunisiensis* MOD 31.J, was found to degrade PAHs such as phenanthrene, pyrene, and benzopyrene^22^.

Among n-alkanes, strain Wilcox degraded n-decane and n-hexadecane as the sole sources of carbon in MSM supplemented with 2.5 M NaCl, but no degradation of methane, hexane, n-eicosane, and n-dotriacontane occurred under similar growth conditions (Table 3). Alkanes are a major component in crude oil and can be biodegraded in the environment by many microorganisms including some halophilic bacteria and archaea such as *Alcanivorax* sp., *Streptomyces albiaxialis, Marinobacter hydrocarbonoclasticus, Haloarcula vallismortis* EH4, and *Haloferax* sp.^16,56–59^. Alkanes are aerobically biodegraded by several pathways. In most cases, degradation is initiated by an alkane hydroxylase system (AlkB) that oxidizes alkanes to corresponding alcohols. This enzyme system (AlkB) consists of three components: the membrane-bound alkane hydroxylase coded by *alkB* that carries out the hydroxylation reaction and two soluble components, rubredoxin (coded by *alkG* gene) and rubredoxin reductase (coded by *alkT* gene), that transfer electrons to the alkane hydroxylase. Generally, C10 to C16 n-alkanes are oxidized by the AlkB system^60,61^. The alcohols produced from n-alkanes undergo further oxidation to corresponding aldehydes and fatty acids by alcohol dehydrogenase and aldehyde dehydrogenase, respectively^7,60–64^. The fatty acids can then be metabolized by beta-oxidation pathway. Our genome analysis predicted the presence of rubredoxin-NAD( +) reductase only, and we did not find the other two components of the alkane hydroxylase system in the genome. However, laboratory studies showed rapid degradation of n-decane and n-hexadecane, suggesting that enzymes other than canonical alkane hydroxylase may be involved the oxidation of alkanes. Strain Wilcox did not degrade methane, n-hexane, n-eicosane, or n-dotriacontane, consistent with the lack of specific genes for these shorter-chain and longer-chain n-alkanes in the genome. The lack of methane metabolisms is consistent with the lack of the key gene methane monooxygenase^65^. Similarly, we did not find *almA* or *ladA*, which encode alkane degrading enzymes for longer-chain n-alkanes^61,62^.

### Conclusion

Although there have been recent studies showing the ability microorganisms to degrade petroleum compounds in various hypersaline environments, not much is known about similar organisms in PW. Hence, studies are needed that explore the ecology, physiology, diversity and hydrocarbon degradation potential of microorganisms native to PW. The isolation and characterization of strain Wilcox suggest that members of the genus *Modicisalibacter* could play an important role in the remediation of saline PW^22,66^. In the present work, we used genomic information to design and query strain Wilcox’s ability to degrade both aliphatic and aromatic compounds in laboratory cultures. For many hydrocarbons, we predicted most of the essential genes needed for a complete metabolism under aerobic conditions. Laboratory studies showed that the strain can efficiently degrade both aliphatic and aromatic compounds at high salinity. Strain Wilcox also harbors genes for the detoxification mechanism of several heavy metals, consistent with presence of a variety of heavy metals in most PW. Furthermore, our bioaugmentation experiments revealed that strain Wilcox is capable of surviving and degrading hydrocarbons present in formation PW without the need for nutrient amendments, suggesting its application to clean-up of PW for beneficial uses.

## Materials and methods

### Chemicals and produced water

Benzene, toluene, ethylbenzene, xylenes, methane, hexane, hexadecane, phenanthrene, and naphthalene were purchased from Sigma-Aldrich, USA. Phenol, biphenyl, phenylacetate, benzoate, n-decane, n-eicosane, and n-dotriacontane were purchased from Fischer Scientific, USA. All other chemicals were of analytical grade and used without further purification. PW used in this study was obtained from the First Wilcox formation (36.03561 N, 97.04483 W), Payne County, OK. The composition of the PW is shown in Supplemental Table S1. The salinity of the PW was 4% (w/v). Total alkalinity and pH were 146 mg/L and 7.42, respectively. Total Dissolved Solids was 146,600 mg/L. The concentrations of magnesium and potassium were around 1,240 mg/L and 698 mg/L, respectively. Low concentrations of several heavy metals were also present.

### Enrichment and isolation of a hydrocarbon degrading bacteria from produced water

The enrichment was performed in 160-ml serum bottles (Wheaton) containing 50 ml of raw PW from First Wilcox formation, Payne County, OK, USA. Bottles were closed with 20-mm Teflon-lined septa and aluminum caps (The West Co). Bottles were injected with 2 µL of each undiluted BTEX compounds and incubated in the dark in an inverted position. Air in the headspace (110 ml headspace) served as the source of oxygen. Biodegradation of added BTEX was monitored using a gas chromatograph (GC) equipped with a flame ionization detector as described before ^40^. Bottles were repeatedly fed BTEX with monthly transfer of 50% of the enrichment culture to a fresh MSM containing 2.5 M NaCl and BTEX (25 – 35 µmol/bottle). After 3–4 months of repeated feeding and transferring a 50% of the culture to fresh medium, a stable enrichment culture was obtained that degraded BTEX in 7 days as the sole carbon and energy sources.

For the isolation of pure culture, the enrichment was plated on to MSM agar plates containing 1 M NaCl. The plates were incubated in a desiccator containing a glass vial with BTEX (2 ml of undiluted BTEX mixture) until colonies appeared (1 week). Well isolated colonies were transferred to serum bottles containing 50 ml of MSM supplemented with 1 M NaCl and 25–35 µmole of each BTEX compound as the sources of carbon. Bottles were closed with Teflon-coated septa and aluminum caps. Headspace sample was withdrawn periodically and monitored for the added BTEX by GC. Bottles that showed rapid BTEX degradation were selected for further characterization and the purity of the isolates was tested by re-plating the culture.

### 16S rRNA -gene analysis of the isolate

An isolate that showed a good BTEX degradation capacity was selected for further study. Genomic DNA was extracted using the Ultra Clean soil DNA kit (Qiagen, Germantown, MD, USA). The isolate was identified by amplifying the 16S rRNA -gene using the 27F and 1492R primers as described before^67^. Amplified PCR product was cleaned using Exo SAP-IT and sequenced at the DNA core facility, Oklahoma State University, Stillwater, OK. The amplified 16S rRNA -gene sequences was analyzed using the National Center for Biotechnology Information (NCBI) database using BLASTN and taxonomically classified using the Silva v138 database^68^.

### Mother inoculum

The isolate was maintained in 1-L bottles with 500 ml of MSM supplemented with 2.5 M NaCl and 25–35 µmole of each BTEX compound as the sole source of carbon and energy. Bottles were closed with Teflon-coated septa and aluminum caps. Headspace was withdrawn periodically and monitored for consumption of the added BTEX. Roughly once every 4 weeks, 50% of the culture was replaced with fresh MSM containing 2.5 M NaCl and BTEX was injected. This culture served as the source of inoculum for all experiments presented in this article unless stated otherwise.

### Genome sequencing, assembly, and annotation

Strain Wilcox was grown in MSM containing 1 M NaCl and 5 mM acetate as the sole source of carbon at 35 °C for 1 week before DNA was extracted. Genomic DNA was extracted using the DNase PowerSoil kit (Qiagen, Germantown, MD, USA). Whole genome shotgun library preparation and sequencing were performed on Illumina HiSeq 4000 by Novogene Corporation (Beijing, China). The 5,747,630 raw 150 bp read pairs were randomly subsampled using Seqtk (https://github.com/lh3/seqtk) to 1.7 million read pairs to achieve an approximate genome coverage of 100X. Reads were then trimmed using Trimmomatic v0.39 with a 4:30 sliding window and minimum length of 36 bp, yielding 1,317,369 read pairs and 169,285 and 53,458 forward and reverse unpaired reads, respectively^69^. All trimmed reads were assembled using SPAdes v3.13 with a minimum approximate coverage of 10X and minimum contig size of 500 bp^70^. The resulting assembly consisted of 43 scaffolds with an N50 180,819, L50 of 7, and total assembly length of 3,584,069 bp. 16S rRNA genes were detected in the assembly using barrnap v0.9 and taxonomy was investigated by submission to Silva database v138^68,71^. Assemblies were submitted to the IMG database^72^ for gene-calling, functional annotation, and to provide public access under genome ID number 2844586914. Un-annotated scaffolds were also deposited to GenBank under accession number WNXF00000000.1. Genes responsible for degradation of aromatic compounds were annotated by Integrated Microbial Genome-expert Review pipeline and the pathways of selective compounds were interpreted using KEGG orthology (KO), TIGRFAM (TIGR), and Clusters of Orthologous Genes (COG) assignments.

### Hydrocarbon degradation experiments

#### Degradation of long-chain alkanes

Flasks (250 ml capacity) containing 100 ml of MSM supplemented with 2.5 M NaCl were prepared and long-chain alkanes such as n-decane, n-hexadecane, n-eicosane or n-dotriacontane was added (2–5 mM) as the sole carbon and energy source. Flasks were inoculated with 2 ml (about 1.2 × 10^6^ CFU) of strain Wilcox culture. Un-inoculated autoclaved flasks containing an n-alkane were setup similarly and served control flasks. Also, inoculated flasks devoid of a substrate (an alkane) were prepared as controls. Flasks were closed with cotton plugs and incubated at 35 °C in the dark. Sample (1 ml) was withdrawn periodically and a 10 × serial dilution was performed and plated on to MSM agar plates containing 1 M NaCl and 5 mM acetate as the carbon source. Plates were incubated at 35 °C for 3 – 5 days and colony forming units (CFU) was determined. All treatments, here and below, were studied in triplicate microcosms.

#### Degradation of short-chain alkanes (methane and hexane)

Degradation of methane was assessed in 160 ml-capacity serum bottles filled with 49 ml of MSM containing 2.5 M NaCl. Bottles were inoculated with 1 ml of strain Wilcox (about 6 × 10^5^ CFU) and closed with rubber stoppers and aluminum crimps. Bottles were injected with 200 µl of pure methane as the sole source carbon and air in the headspace served as the source of oxygen. Un-inoculated and autoclaved bottles containing methane were set up similarly as controls. Bottles were incubated upside-down at 35 °C in the dark. Headspace sample was withdrawn periodically, and methane was quantified using a Hewlett Packard Agilent 6890 N GC (Agilent Technology) equipped with a TCD detector as described before^41^.

Biodegradation of hexane was assessed in 160 ml-capacity serum bottles filled with 49 ml of MSM containing 2.5 M NaCl. Bottles were amended with 5 mM hexane as the sole carbon and energy source and sealed with rubber septa and aluminum caps. Bottles were inoculated with 1 ml of strain Wilcox (about 6 × 10^5^ CFU). Un-inoculated autoclaved bottles containing hexane were set up similarly as controls. Also, bottles inoculated with strain Wilcox but lacking the substrate (hexane) were prepared similarly and served as controls. Bottles were incubated upside-down at 35^o^ C in the dark. Culture sample (1 ml) was withdrawn periodically and a 10 × serial dilution was performed and plated on MSM agar plates containing 1 M NaCl and 5 mM acetate as the carbon source. CFU was determined after 3–5 days of incubation.

#### Degradation of BTEX

Degradation of BTEX was tested in 160-ml-capacity serum bottles filled with 49 ml of MSM containing 2.5 M NaCl. Bottles were closed with Teflon-coated septa and aluminum caps and injected with 2 µl of undiluted BTEX (each 25–35 µmol/bottle) as the sole carbon and energy source. Bottles were inoculated with 1 ml (roughly 6 × 10^5^ CFU) of strain Wilcox. Un-inoculated and autoclaved bottles containing BTEX were set up similarly as controls. Bottles were incubated upside-down at 35 °C in the dark. The headspace was withdrawn periodically, and degradation of BTEX was monitored using GC as mentioned above.

#### Salinity and BTEX degradation

The effect of salinity on BTEX degradation was determined in 160-ml serum bottles containing 48-ml of MSM supplemented with 0.016, 0.5, 1.0, 2.0, 3.0, 4.0 or 5.0 M NaCl. Bottles were closed with Teflon-coated septa and aluminum caps. Bottles were injected with 2 µl of undiluted BTEX (each 25–35 µmole) and inoculated with 2 ml (roughly 2 × 10^6^ CFU) of strain Wilcox. Un-inoculated and autoclaved bottles containing BTEX were set up similarly as controls. Bottles were incubated upside-down at 35 °C in the dark. Headspace (100 µL) was withdrawn periodically and degradation of the BTEX was measured using GC.

#### Degradation of benzoate, biphenyl, and phenylacetate

Flasks (250 ml capacity) containing 100 ml MSM supplemented with 2.5 M NaCl and 1–2 mM benzoate, biphenyl, or phenylacetate as the sole source of carbon were set-up. Flasks were inoculated with 2 ml of strain Wilcox (5 × 10^4^ to 1.2 × 10^6^ CFU) and incubated at 35 °C. Un-inoculated and autoclaved flasks containing benzoate, biphenyl, or phenylacetate were prepared similarly and served as controls. Also, autoclaved flasks inoculated with strain Wilcox but lacking a substrate served as controls. Flasks were closed with cotton plugs and incubated at 35°C in the dark. To monitor benzoate degradation, culture (1 ml) was withdrawn periodically and centrifuged at 9,391 × g for 10 min. The cell-free supernatant was appropriately diluted in nano pure water and benzoate concentration was monitored by measuring absorption at 223 λ using the Ultraspec 2000 UV/Visible spectrophotometer. To monitor the growth of strain Wilcox on biphenyl or phenylacetate as the sole carbon source, culture sample (1 ml) was withdrawn periodically and a 10 × serial dilution was performed and plated on MSM agar plates containing 1 M NaCl and 5 mM acetate as the carbon source. Plates were incubated at 35 °C for 3–5 days and CFU was determined.

#### Degradation of phenol

Degradation of phenol was determined in 160-ml serum bottles filled with 49 ml of MSM supplemented with 2.5 M NaCl. Bottles were amended with 2 mM phenol as the sole carbon and energy source and inoculated with 1 ml of strain Wilcox culture (roughly 6 × 10^5^ CFU). Bottles were closed with Teflon-coated septa and aluminum caps. Un-inoculated autoclaved flasks containing phenol were set up similarly as controls. Also, inoculated flasks devoid of phenol were set up similarly and served as controls. Bottles were incubated at 35 °C in the dark. Culture sample (1 ml) was withdrawn periodically and a 10 × serial dilution was performed and plated on MSM agar plates containing 1 M NaCl and 5 mM acetate as the carbon source. Plates were incubated at 35 °C for 3–5 days and CFU was determined.

### Bioaugmentation potential of strain Wilcox

Serum bottles (160-ml capacity) were filled with 48-ml of formation PW from Payne County (nothing other than BTEX compounds were added). Bottles were closed with Teflon-coated septa and aluminum caps and sterilized by autoclaving. Bottles were amended with 100–200 µmol of each BTEX compound combined. Also, bottles were prepared similarly and amended with 100–200 µmoles of B, T, E or X (as individual compounds). All bottles were inoculated with 2 ml of strain Wilcox culture (roughly 2 × 10^6^ CFU). Autoclaved un-inoculated bottles amended with 100–200 µmol of each B, T, E, and/or X compound (s) were prepared similarly and served as controls. 100 µL headspace was withdrawn periodically to determine degradation of the BTEX compounds using GC. The composition of the formation PW can be found in Supplemental Table S1.

## Supplementary Information

Supplementary Information

## Data Availability

Assemblies were submitted to the IMG database for gene-calling, functional annotation, and to provide public access under genome ID number 2844586914. Un-annotated scaffolds were also deposited to GenBank under accession number WNXF00000000.1.
