# Supplementary material for: Isolation and characterization of a halophilic Modicisalibacter sp. strain Wilcox from produced water
Source: Sci Rep. 2021 Mar 25;11:6943. doi: 10.1038/s41598-021-86196-0 (PMC7994583; doi:10.1038/s41598-021-86196-0)
Supplement: Supplementary file 1 — Supplementary Information [file 41598_2021_86196_MOESM1_ESM.pdf]

Supplementary Material

Isolation and Characterization of a Halophilic *Modicisalibacter* sp. strain Wilcox from Produced Water

William S. Marsh[1], Brenden W. Heise[1], Mark J. Krzmarzick[2]; Robert W. Murdoch[3,4], and Babu Z. Fathepure[1]*.

Department of Microbiology and Molecular Genetics[1], Civil and Environmental Engineering[2], Oklahoma State University, Stillwater, OK 74078 USA; Center for Environmental Biotechnology[3], University of Tennessee, Knoxville, TN 379963; Battelle Memorial Institute[4], Columbus, OH4

***Corresponding author: babu.fathepure@okstate.edu**

Supplemental Tables

Supplemental Table S1. The Wilcox Produced Water Characteristics

Test	Concentration	Unit
Alkalinity	146	mg/L
Bicarbonate	146	mg/L
Bromide	301	mg/L
Chloride	76, 700	mg/L
Cyanide	[a]BPQL	mg/L
Flouride	BPQL	mg/L
Ortho-phosphate	0.26	mg/L
pH	7.41	
Total Nitrogen	74.2	mg/L
Total Suspended Solids	84.7	mg/L
Conductivity	250, 200	umhoc/cm
Nitrate	BPQL	mg/L
Sulfate	251	mg/L
Total Dissolved Solutes	146,600	mg/L
Total Organic Carbon	0.632	mg/L
Total Kjeldahl Nitrogen	74.2	mg/L
Oil & Grease	BPQL	mg/L
Aluminum	BPQL	mg/L
Arsenic	0.0492	mg/L
Barium	2.73	mg/L
Boron	6.79	mg/L
Calcium	7, 120	mg/L
Chromium	BPQL	mg/L
Copper	0.269	mg/L
Iron	0.317	mg/L
Lead	BPQL	mg/L
Lithium	4.48	mg/L
Magnesium	1, 240	mg/L
Manganese	0.303	mg/L
Nickel	BPQL	mg/L
Potassium	698	mg/L
Selenium	0.0006	mg/L
Silica	20.5	mg/L
Silver	BPQL	mg/L
Sodium	42, 600	mg/L
Sodium Absorption Ratio	123	
Strontium	445	mg/L
Zinc	BPQL	mg/L

[a]BPQL = Below Practical Quantitation Limit

Supplemental Table S 2. *Modicisalibacter* sp. strain Wilcox genes predicted to encode proteins involved in metabolism of hydrocarbons. Functional annotation used to derive predictions: KO= Kyoto Encyclopedia of Genes and Genomes Orthology, COG= Cluster of Orthologous Genes, TIGR= TIGRFAM.

**IMG Gene ID**	**Substrate (s)**	**Functional Annotation**	**Description**
2844586937	Biphenyls	KO:K00462	biphenyl-2,3-diol 1,2-dioxygenase [EC:1.13.11.39]
2844586938	Biphenyls	KO:K08690	cis-2,3-dihydrobiphenyl-2,3-diol dehydrogenase [EC:1.3.1.56]
2844586939	Benzene	KO:K18088	biphenyl 2,3-dioxygenase ferredoxin reductase subunit [EC:1.18.1.3]
2844586941	Biphenyls	KO:K15750	biphenyl 2,3-dioxygenase beta subunit [EC:1.14.12.18]
2844586942	Biphenyls	KO:K08689	biphenyl 2,3-dioxygenase alpha subunit [EC:1.14.12.18]
2844586958	Catechol	COG2153	Predicted N-acyltransferase, GNAT family
2844587095	Alkanes	KO:K00138	aldehyde dehydrogenase [EC:1.2.1.-]
2844587430	Lignin-like compounds	COG3384	Aromatic ring-opening dioxygenase, catalytic subunit, LigB family
2844587495	Salicylate	KO:K00480	salicylate hydroxylase [EC:1.14.13.1]
2844587528	Alkanes	KO:K05297	rubredoxin-NAD+ reductase [EC:1.18.1.1]
2844588030	Benzoate	COG0596	Pimeloyl-ACP methyl ester carboxylesterase
2844588354	Catechol	KO:K00632	acetyl-CoA acyltransferase [EC:2.3.1.16]
2844588659	Protochatuate	KO:K00448	protocatechuate 3,4-dioxygenase, alpha subunit [EC:1.13.11.3]
2844588660	Protochatechate	KO:K00449	protocatechuate 3,4-dioxygenase, beta subunit [EC:1.13.11.3]
2844588668	Phenylacetate	KO:K00626	acetyl-CoA C-acetyltransferase [EC:2.3.1.9]
2844588669	Catechol	KO:K01040	glutaconate CoA-transferase, subunit B [EC:2.8.3.12]
2844588670	Catechol	KO:K01039	glutaconate CoA-transferase, subunit A [EC:2.8.3.12]
2844588671	Protochatechuate	KO:K01857	3-carboxy-cis,cis-muconate cycloisomerase [EC:5.5.1.2]
2844588678	Phenylacetate	KO:K00074	3-hydroxybutyryl-CoA dehydrogenase [EC:1.1.1.157]
2844588712	3-maleylpyruvate	KO:K01800	maleylacetoacetate isomerase [EC:5.2.1.2]
2844589229	Alkanes	COG0604	NADPH:quinone reductase or related Zn-dependent oxidoreductase
2844589416	Alkanes	KO:K03386	peroxiredoxin (alkyl hydroperoxide reductase subunit C) [EC:1.11.1.15]
2844589672	Homogentisate	KO:K00451	homogentisate 1,2-dioxygenase [EC:1.13.11.5]
2844589728	Phenylacetate	KO:K02613	ring-1,2-phenylacetyl-CoA epoxidase subunit PaaE
2844589730	Phenylacetate	KO:K02611	ring-1,2-phenylacetyl-CoA epoxidase subunit PaaC [EC:1.14.13.149]
2844589731	Phenylacetate	KO:K02610	ring-1,2-phenylacetyl-CoA epoxidase subunit PaaB
2844589732	Phenylacetate	KO:K02609	ring-1,2-phenylacetyl-CoA epoxidase subunit PaaA [EC:1.14.13.149]
2844589733	Phenylacetate	KO:K01912	phenylacetate-CoA ligase [EC:6.2.1.30]
2844589734	Phenylacetate	COG0183	Acetyl-CoA acetyltransferase
2844589737	Phenylacetate	KO:K15866	2-(1,2-epoxy-1,2-dihydrophenyl)acetyl-CoA isomerase [EC:5.3.3.18]
2844589738	Phenylacetate	KO:K01692	enoyl-CoA hydratase [EC:4.2.1.17]

2844589751	Catechol	TIGR02534	muconate and chloromuconate cycloisomerases
2844589752	Catechol	COG4948	L-alanine-DL-glutamate epimerase or related enzyme of enolase superfamily
2844589753	Catechol	KO:K03464	muconolactone D-isomerase [EC:5.3.3.4]
2844589754	Catechol	KO:K03381	catechol 1,2-dioxygenase [EC:1.13.11.1]
2844589755	Benzoate	KO:K05549	benzoate/toluate 1,2-dioxygenase alpha subunit [EC:1.14.12.10 1.14.12.-]
2844589756	Benzoate	KO:K05550	benzoate/toluate 1,2-dioxygenase beta subunit [EC:1.14.12.10 1.14.12.-]
2844589757	Benzoate	KO:K05784	benzoate/toluate 1,2-dioxygenase reductase subunit [EC:1.18.1.-]
2844589758	Benzoate	COG1028	NAD(P)-dependent dehydrogenase, short-chain alcohol dehydrogenase family
2844589848	4-hydroxybenzoate	KO:K00481	p-hydroxybenzoate 3-monooxygenase [EC:1.14.13.2]
2844589969	Catechol	KO:K00626	acetyl-CoA C-acetyltransferase [EC:2.3.1.9]
2844590194	Aromatics	COG0633	Ferredoxin

Supplemental Table S 3. *Modicisalibacter* sp. strain Wilcox genes predicted to encode proteins involved in heavy metal resistance. Functional annotation used to derive predictions: KO= Kyoto Encyclopedia of Genes and Genomes Orthology, COG= Cluster of Orthologous Genes, TIGR= TIGRFAM.

**IMG Gene ID**	**Metal**	**Functional Annotation**	**Description**
2844587032	As	KO:K03325	arsenite transporter, ACR3 family
2844587034	As	KO:K00537	arsenate reductase [EC:1.20.4.1]
2844587328	Cd, Co, Zn	KO:K16264	cobalt-zinc-cadmium efflux system protein
2844587374	Mn	KO:K02075	zinc/manganese transport system permease protein
2844587477	Hg	KO:K00520	mercuric reductase [EC:1.16.1.1]
2844587480	Hg	TIGR02053	mercury(II) reductase
2844587740	As	KO:K01551	arsenite-transporting ATPase [EC:3.6.3.16]
2844587781	Cu	KO:K01533	Cu2+-exporting ATPase [EC:3.6.3.4]
2844587847	Cu	KO:K17686	Cu+-exporting ATPase [EC:3.6.3.54]
2844587870	Mn	COG1971	Putative Mn2+ efflux pump MntP
2844588193	Zn	KO:K09823	Fur family transcriptional regulator, zinc uptake regulator
2844588553	Zn	KO:K16074	zinc transporter
2844588586	Cd, Zn, Pb	KO:K01534	Cd2+/Zn2+-exporting ATPase [EC:3.6.3.3 3.6.3.5]
2844589668	As	KO:K03893	arsenical pump membrane protein
2844589669	As	KO:K00537	arsenate reductase [EC:1.20.4.1]
2844589689	Mn, Zn	TIGR01525	heavy metal translocating P-type ATPase
2844589846	Cu	COG2132	Multicopper oxidase with three cupredoxin domains (includes cell division protein FtsP and spore coat protein CotA)
2844589924	Mn, Co	KO:K06189	magnesium and cobalt transporter
2844590090	Co	KO:K03795	sirohydrochlorin cobaltochelatase [EC:4.99.1.3]

Supplemental Figures

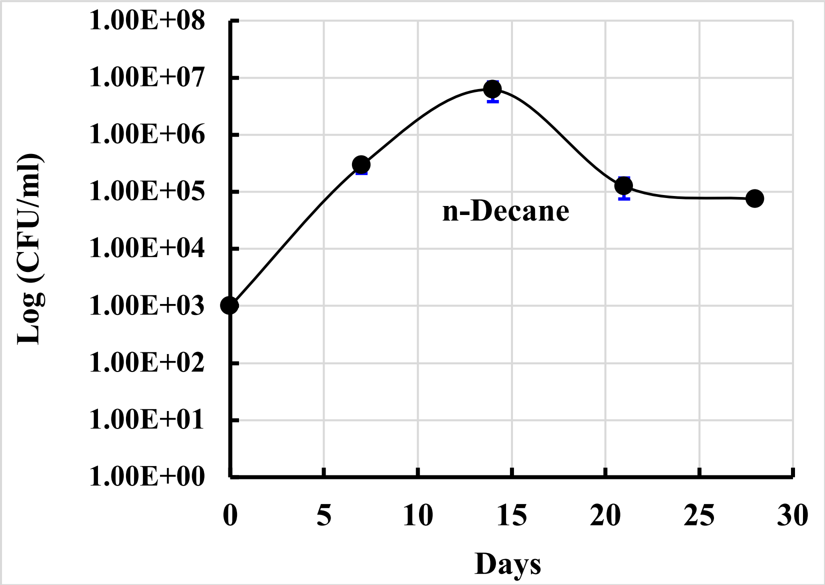

Supplemental Figure S 1. Growth of strain Wilcox on n-decane as the sole carbon source. Flasks containing 100 ml of MSM supplemented with 2.5 M NaCl and 2 mM n-decane as the sole source of carbon were inoculated with strain Wilcox. Growth of the strain was monitored as CFU/ml by plating the culture at time points on MSM agar plates containing 1 M NaCl and 5 mM acetate. No increase in CFU occurred in control flasks devoid of added n-decane (data not shown). Error bars indicate ±1 standard deviation of triplicate bottles (n=3).

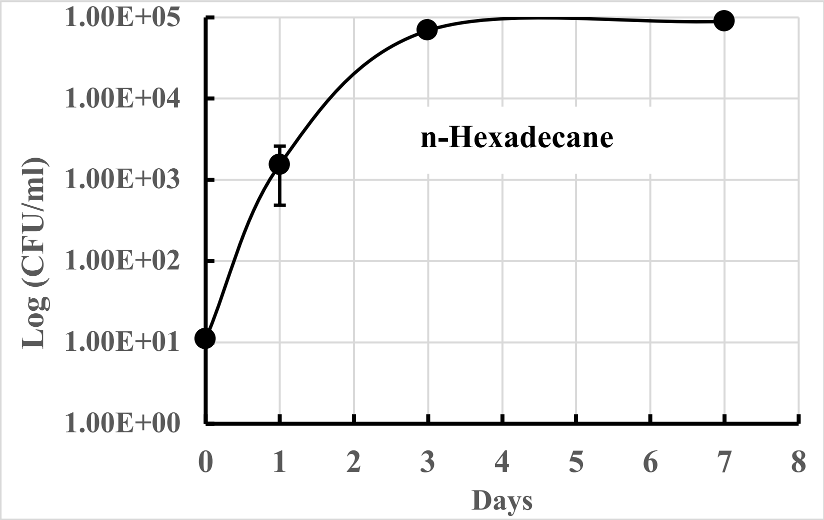

Supplemental Figure S 2: Growth of strain Wilcox on n-hexadecane as the sole source of carbon. Flasks containing 100 ml of MSM supplemented with 2.5 M NaCl and 5 mM n-hexadecane as the sole source of carbon were inoculated with strain Wilcox. Growth of the strain was monitored as CFU/ml by plating the culture at time points on MSM agar plates containing 1 M NaCl and 5 mM acetate. No increase in CFU occurred in control flasks devoid of added n-hexadecane (data not shown). Error bars indicate ±1 standard deviation of triplicate bottles (n=3).

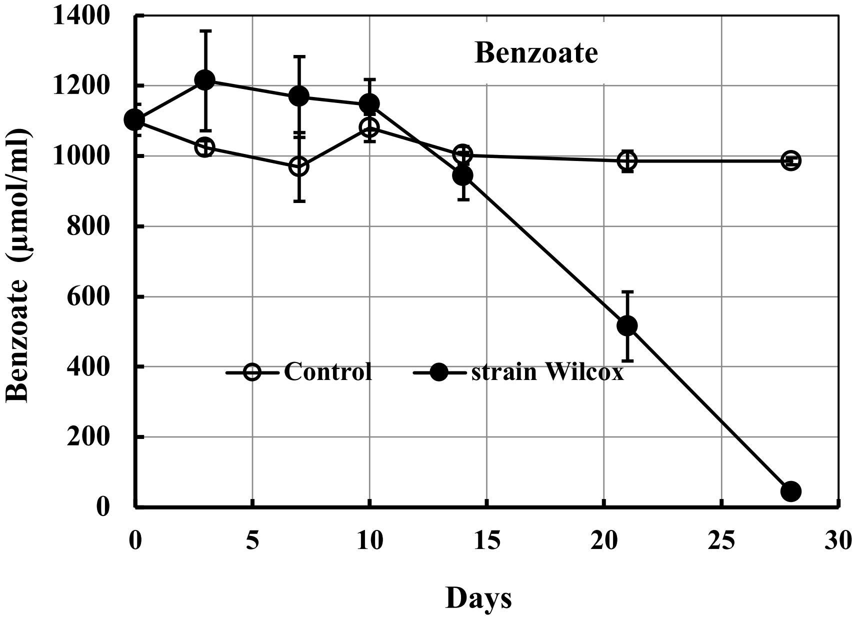

Supplemental Figure S 3: Biodegradation of benzoate by strain Wilcox. Flasks containing 100 ml of MSM supplemented with 2.5 M NaCl and 1mM benzoate as the sole source of carbon were inoculated with strain Wilcox. Un-inoculated control flasks were setup similarly. Degradation of benzoate was monitored by measuring absorption at λ 223 in cell-free supernatants using UV-VIS spectrophotometer. Error bars indicate ±1 standard deviation of triplicate bottles (n=3).

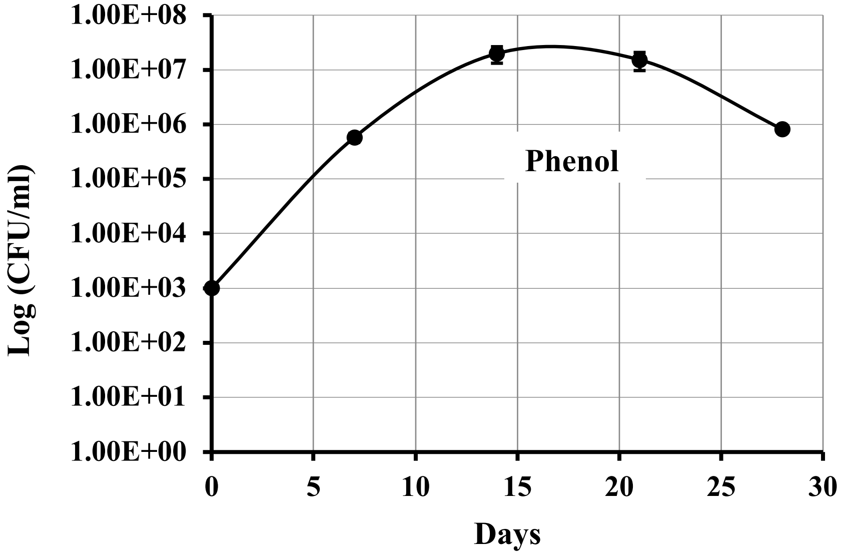

Supplemental Figure S 4. Degradation of phenol by strain Wilcox. Serum bottle (160 ml-capacity) containing 49 ml of MSM supplemented with 2.5 M NaCl and 2 mM phenol as the sole source of carbon were inoculated with strain Wilcox. Bottles were closed with rubber septa and aluminum crimps. Growth of the strain was monitored as CFU/ml by plating the culture at time points on MSM agar plates containing 1 M NaCl and 5 mM acetate. No increase in CFU occurred in control flasks devoid of added phenol (data not shown). Error bars indicate ±1 standard deviation of triplicate bottles (n=3).

\

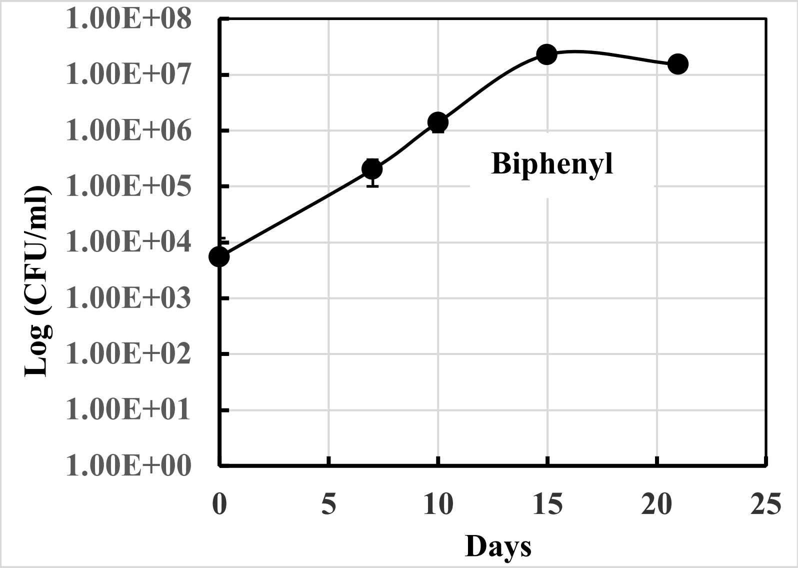

Supplemental Figure S 5. Degradation of biphenyl by strain Wilcox. Flasks containing 100 ml of MSM supplemented with 2.5 M NaCl and 2 mM biphenyl as the sole source of carbon were inoculated with strain Wilcox. Growth of the strain was monitored as CFU/ml by plating the culture at time points on MSM agar plates containing 1 M NaCl and 5 mM acetate. No increase in CFU occurred in control flasks devoid of added biphenyl (data not shown). Error bars indicate ±1 standard deviation of triplicate bottles (n=3).

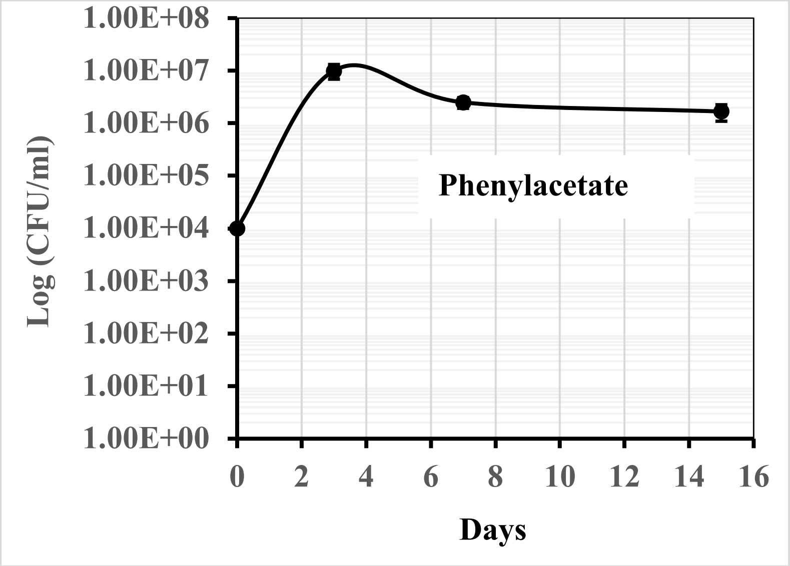

Supplemental Figure S 6. Degradation of phenylacetate by strain Wilcox. Flasks containing 100 ml of MSM supplemented with 2.5 M NaCl and 2 mM phenylacetate as the sole source of carbon were inoculated with strain Wilcox. Growth of the strain was monitored as CFU/ml by plating the culture at time points on MSM agar plates containing 1 M NaCl and 5 mM acetate. No increase in CFU occurred in control flasks devoid of added phenylacetate (data not shown). Error bars indicate ±1 standard deviation of triplicate bottles (n=3).
